# Household Management

**Published:** 1916-06-03

**Authors:** 


### THE MATRONS' AND SISTERS' DEPARTMENT.

### HOUSEHOLD MANAGEMENT.

### THE LITTLE STORE SNARE.

The secret of dealing economically with large reserves in every branch of household management lies in a good system of stock-taking. The first problem to deal with is the quantity of goods to keep in stock. This is to be settled partly by the accommodation available, partly by the weekly consumption of the articles in question, and partly by the conditions under which the goods are best purchasable. Too much time and consideration cannot be devoted in the first instance to these points, and the supply of various goods ought to be co-ordinated as far as possible, so that new stocks may be received only at stated intervals without risk of things running short. The housekeeper will have to experiment again and again before she reaches a reliable estimate of the amount of goods she must have in stock to meet requirements for the week or the month or the quarter, whichever time of renewal be found most convenient. The bins must be so arranged that new supplies are not dumped down upon the remains of the old, that old bottles of fruit, pickles, etc., do not get pushed to the back of the shelf to moulder while the new consignment is worked through, and that over-estimates of the quantities required are promptly detected. The pitfalls which beset the store-room are beyond enumeration, but they rest mainly upon faulty construction and faulty estimates.

In practice it is often found that the great impediment to forming accurate estimates of the weekly cost of boarding a household lies in the store-room. Certain articles, such as tea, coffee, sugar, and cereals, can be bought at a reduction in bulk. For these in a slipshod household there is no weekly estimate of cost such as tradesman or contractor furnishes in regard to meat, fish, bread, milk, etc. Once a quarter, or at varying intervals when things "run out," a box of tea enters the accounts, or a few hundredweight of groceries. The housekeeper rests satisfied that these goods ought to last a certain length of time. She takes what is wanted daily from the store-cupboard, but she shirks the trouble of requisitioning as she ought to do from her own stores as carefully as she requisitions from the tradesmen. We are speaking now of such smallish establishments as have no steward, perhaps no regular housekeeper. The economies effected by buying in bulk are therefore often nullified by loss of control. When the housekeeper thinks it can serve no good purpose to estimate exactly how much tea, for instance, is used every week, but is content with using "as little as possible," the reins lie slack and waste is the inevitable result. The housekeeper who requisitions the exact quantity of stores every day, even if it be from herself, does far more than check waste in the store-room. She is laying the foundation for that exact knowledge of what is required for her household, be it a hundred or be it ten, wherein lies the secret of economy.

